# Normalized Method for Land Surface Temperature Monitoring on Coastal Reclaimed Areas

**DOI:** 10.3390/s19224836

**Published:** 2019-11-06

**Authors:** Bahaa Mohamadi, Shuisen Chen, Timo Balz, Khansa Gulshad, Stephen C. McClure

**Affiliations:** 1 State Key Laboratory of Information Engineering in Surveying, Mapping and Remote Sensing (LIESMARS), Wuhan University, Wuhan 430072, China; bh.mo@whu.edu.cn (B.M.); balz@whu.edu.cn (T.B.); khansag2@gmail.com (K.G.); stephen.c.mcclure@hotmail.com (S.C.M.); 2 Key Lab of Guangdong for Utilization of Remote Sensing and Geographical Information System, Guangdong Open Laboratory of Geospatial Information Technology and Application, Guangdong Engineering Technology Center for Remote Sensing Big Data Application, Guangzhou Institute of Geography, Guangzhou 510070, China

**Keywords:** coastal environment, land reclamation, land surface temperature (LST), pearl river estuary, urban heat islands (UHI)

## Abstract

The temporal analysis of land surface temperature (LST) has generally been studied using data from the same season, as temperature varies greatly over time. However, the cloud cover in thermal remotely sensed images and the coarse resolution of passive sensor system significantly limits data availability of same season for comparative temporal analysis in many parts of the world. To address this problem, we propose a new method for temporal monitoring of surface temperature based on LST normalization (LSTn); deploying the average open water temperature to normalize LST when monitoring temporal change in the surface temperature of newly coastal reclaimed areas. This method was applied in the Lingding Bay area, Guangdong Province, Southern China. Original LST and LSTn values were calculated for years 1987, 1997, 2007, and 2017. In contrast to the original LST, results show that LSTn can reduce seasonal variability when monitoring temporal change in surface temperatures. Additionally, LSTn revealed pronounced differences between the temperature of impervious surfaces and other land cover types. This method offers more robust detection of surface urban heat islands than original LST in newly developed coastal areas.

## 1. Introduction

The wide coverage, cost efficiency, and time series of observations over the entire globe are the main advantage of remote sensing data, which allows the usage of land surface temperature (LST) as a variable to study rapid urbanization, destruction of vegetated areas, and climate change at either local or global scale [1]. LST increased in urban areas of many countries of the world, due to land cover changes from natural environments into urban impervious surfaces (UIS), resulting in the creation of new surface urban heat islands (SUHI) [2]. SUHIs induce thermal stress on human bodies especially in summer seasons, and increase the intensity and duration of heat waves [3], which could result in rising death rates during heat waves in coastal areas [4]. Hence, monitoring surface temperature in urban areas might help planners abate and mitigate potential thermal stress threats to people living and working in those areas.

Monitoring temporal LST and SUHI changes is challenging due to the seasonal variance in temperature, and even daily changes within the same season. Some studies revealed LST changes based on only a single image per period [5,6], raising doubts on their results reliability. While some other studies sacrificed the time series analysis by compose images of many years—sometimes 30 years divided into two periods—to do a reliable average of LST values [7]. Other efforts have been made to fuse coarse spatial resolution and high temporal resolution data such as MODIS (Moderate Resolution Imaging Spectroradiometer), with moderate spatial resolution and low temporal resolution data such as Landsat and ASTER (Advanced Spaceborne Thermal Emission and Reflection Radiometer) data, to overcome cloud cover and long time span of moderate spatial resolution data problems. However, this solution in many cases is inapplicable as it is time intensive demanding large computing resources. While, taking the advantage of using MODIS high temporal resolution data alone might be useful to select appropriate images for LST processing in large study areas, but it will not be helpful in studying small areas of interest such as coastal reclaimed areas because of the data coarse spatial resolution. 

Land reclamation is a well-known solution for land augmentation in coastal areas under pressure from population increases, rapid urbanization, and economic development [8,9]. Land reclamation, however, can result in unanticipated outcomes as it changes the thermodynamic condition of the coastal area’s surface, leading to the increase in LST, and results in the creation of SUHIs [10]. As the reclaimed land is a dynamic environment, the study of spatial and temporal changes of SUHI on these areas faces additional difficulties due to continuous land cover change that alters land surface temperature rapidly over time. Hence, reclaimed lands in regular cloudy weather need an LST temporal comparison method, independent of seasonal surface temperature variations for SUHIs detection. Therefore, we have developed a new normalized method to minimize the temporal variance of LST in coastal areas—especially on coastal reclaimed areas—for LST monitoring and SUHI extraction. 

Several researchers used normalization methods—especially the min–max feature scaling—to represent LST changes over time to support their findings [5,11]. Nevertheless, to the best of our knowledge, previous studies seldom used any normalization method for cross-season comparisons or SUHI extraction. We call our method the land surface temperature normalization method (LSTn), which is based on the average of water surface temperature for time-varying comparisons and SUHI extraction. We selected the average temperature of the water surface for LST normalization because water was the original land cover in the area of interest before the reclamation process. Therefore, we estimate the land surface temperature changes based on the average of the original land cover LST.

We selected reclaimed areas of Lingding Bay, Southern China, to apply the proposed method, because of the rapid reclamation processes and high urbanization rates occurring there since the late 1970s. Besides, the area of Lingding Bay has a dense cloud cover during the year with rare available moderate spatial resolution images suitable for LST processing. Previous studies estimated LST in the Lingding Bay coastal area as a part of the Pearl River Estuary (PRE) region, depending on single image per period due to the lack of data in this study area [4,12,13,14]. These studies revealed a positive correlation between LST and urban impervious surfaces (UIS), and negative relationship between LST and vegetation. Since these findings have been confirmed in the literature, we correlated the land cover type and UIS results from our proposed method to validate the applicability of LSTn for temporal monitoring and SUHI extraction in the study area.

The rest of this paper is as follows, Section 2 describes the methodology and background of the proposed model. Section 3 presents the results of the applied method in Lingding Bay and shows advantages and disadvantages of the LSTn method. Section 4 discusses the results of the method in Lingding Bay and its correlations to the literature. Conclusions are drawn in Section 5.

## 2. Materials and Methods

**Figure 1:**
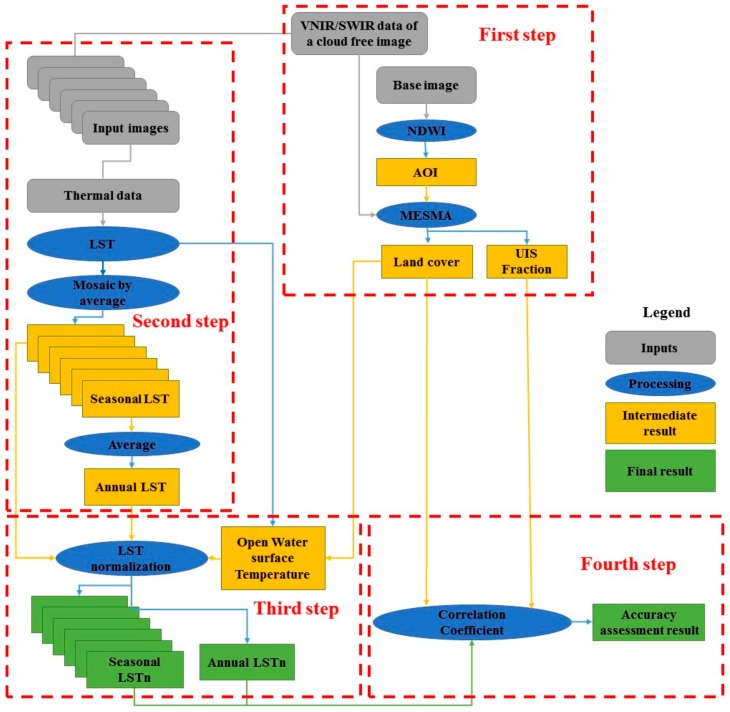
Workflow of the LSTn method.

Figure 1 illustrates the workflow of the LSTn method. In the first step, we define the area of interest (AOI), by applying the normalized difference water index (NDWI) on the first available Landsat image of the target study area. Reclaimed areas are extracted by subtracting the NDWI of a recent image from the NDWI of the first Landsat image. A single free cloud image is selected for each studied year to classify land cover and extract the urban impervious surfaces (UIS) fraction using the multiple endmember spectral mixture analysis (MESMA) technique, based on the visible near-infrared and short wave infrared (VNIR/SWIR) wavelength bands.

In the second step, we estimate the land surface temperature (LST) for all available images after isolating cloud cover pixels from the scenes. Results for each season in a studied year are averaged to map seasonal LST. Another averaging estimates the annual LST from seasonal results. The third step consists of a normalization of seasonal and annual LST averages (LSTn) based on the average of open water surface temperature. The fourth and last step correlates LSTn to land cover and the UIS fraction validates the LSTn results for temporal monitoring of surface temperature and SUHIs extraction. 

In this method, we did not use LST outputs from passive microwave sensors, because these products are still at a coarse resolution unlike those products extracted from thermal infrared remote sensing data [15], which are inapplicable in the relatively small coastal reclaimed areas. We therefore, used thermal remote sensing data available at various spatial scales for assessing SUHI in reclaimed areas and other relatively small coastal environments. Details of the LSTn method application in the Lingding Bay are presented in the following subsections.

### 2.1. Study Area and Data Collection 

**Figure 2:**
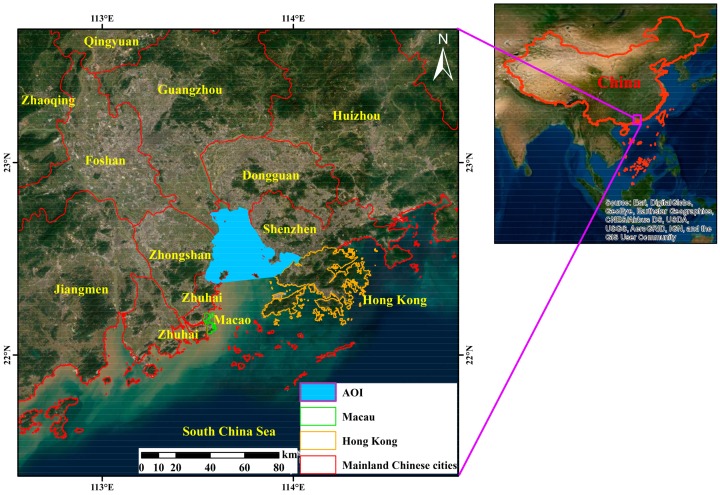
Area of interest (AOI) in southern China as the LSTn method test area.

The Lingding Bay is the main outlet of the Pearl River to the South China Sea. It is located in the south of Guangdong Province, an area of more than 1000 km^2^, with a sub-tropical monsoon climate. The coastal area is under an intense pressure from land reclamation; many parts are considered artificial environments given the extent of recent economic development. Land reclamation in this area was a solution for the local governments to maintain the high population density of the Pearl River Estuary (PRE) region, exceeding 1200/km^2^, related to the rapid evolution of industrialization in the Pearl River Delta since the late 1970s [16]. Figure 2 presents the study area consisting of the coastal zone of Shenzhen, Dongguan, Guangzhou, Zhongshan, and the north of Zhuhai (hereafter, it will be referred to as Zhuhai) districts in Guangdong Province.

Figure 2 illustrates the area of interest (AOI) in light blue, mainland Chinese cities have red borders, Hong Kong have electron gold borders, and Macau within green borders. To define the reclaimed lands in Lingding Bay as the area of interest (AOI), we used the first available Landsat Multispectral Scanner System MSS image in the PRE, acquired on December 23, 1973, to extract the water body as AOI in 1973. Table 1 illustrates all Landsat images used in this study with their respective seasons and data types. Among all images, we employed four cloud free Landsat images separated by about a decade, defined the areas reclaimed since 1973 (starred in Table 1). 

As shown in Table 1, each period has data within a year buffer before and after each year period. Because, cloud free Landsat data are rare in this study area during a single year period. That decision was an effort to extract a very reliable LST average of all seasons during each period, rather than depending on a few sets of images during a single year. We combine the data of spring and autumn seasons together in one set for the same reason mentioned above. Winter season images are those acquired in December, January, and February. Images sensed in June, July, and August are corresponding to the summer season. While, spring/autumn images are those acquired in March, April, and May representing the Spring season, and September, October, and November images of Autumn season. Images from less than 60% cloud cover were collected for this case study. However, for the summer of 1987 period and winter of 1997 period there was only one image per season for the three corresponding years of the study period, as shown in Table 1. 

### 2.2. Reclaimed Areas Extraction

We used the normalized difference water index (NDWI) on the Landsat MSS image to extract the Lingding Bay water body in 1973. This method uses the fact that water shows high reflectance in the green light wavelength and its low reflectance in the near-infrared wavelength using the expression [18]

(1) $$NDWI=\frac{G-NIR}{G+NIR}$$

where G is a band that reflects green light and NIR is the near-infrared band. In the next step, we converted the extracted water surface of Lingding Bay to a vector layer in a base map, and used the Landsat 8 image collected on 7 February 2016 to extract the reclaimed area extent since 1973. The extracted reclaimed lands were defined as the study area of interest for monitoring LST and SUHI in Lingding Bay. 

The Fast Line-of-sight Atmospheric Analysis of Spectral Hypercubes (FLAASH) module was applied to optical bands of the four Landsat images, starred in Table 1, to correct atmospheric effects. Then, we used VIPERTools add-on in the ENVI 5.1 software environment to classify land cover in the study area based on multiple endmember spectral mixture analysis (MESMA) technique following the guideline of the add-on tool [19]. MESMA has the advantage of decomposing each pixel using a combination of representational endmembers [20], and it applies by running several models for each pixel to select the best fit one with minimum RMSE and has successfully been tested to map the majority of image pixels with two-endmember models only [19]. 

**Figure 3:**
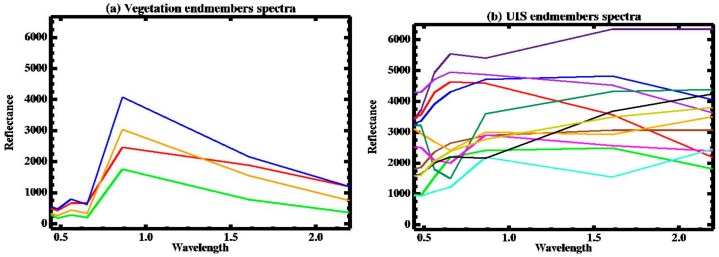
Spectra used to classify land cover in this study; (**a**) Vegetation endmembers spectra; (**b**) UIS endmembers spectra.

VIPERTools is a series of steps started from collecting spectra from the image to end by mapping the land cover based on single or multi-spectral mixture analysis technique. In short, this process started by collecting different pixel spectra for five land cover types (water, shallow water, vegetation, bare soil, and impervious surfaces) to choose the purest pixels in each group as endmembers. VIPERTools has the advantage of using different approaches to select the optimal endmembers for each chosen feature (the Count-based Endmember Selection (CoB), the Endmember Average RMSE (EAR), and the Minimum Average Spectral Angle (MASA)). The optimum selection of endmembers had the highest in-CoB value (the total number of spectra modeled within the class) and the lowest EAR and MASA [19]. Selected endmembers for each land cover classification were applied iteratively in a linear spectral unmixing analysis process using VIPERTools to define the best-fit spectra to map land cover types based on the multi-spectral analysis technique of MESMA. We processed the MESMA analysis two times; first, to map the five land cover types mentioned above. Second, we processed it with two-endmember models—one of UIS endmember spectra, and other of vegetation endmember spectra—to extract UIS composition inside each pixel representing the reclaimed areas. Spectra presented in Figure 3 for UIS and vegetation were used to produce the two multi-endmembers classification image for each studied period.

Figure 3a, shows vegetation endmember spectra used in the second MESMA processing with high reflectance in the NIR wavelength, in comparison to other Landsat wavelengths; especially the red band. Variant spectra reflected by UIS endmembers represent different UIS materials inside urban areas, seen in Figure 3b. Results of the second MESMA procedure include values of UIS and vegetation combined with shade effects inside each pixel. Hence, Equation (2) was used to calculate urban impervious surfaces composition inside each pixel by isolating the shade fraction results

(2) $$C=\frac{U}{V+U}\;\times 100$$

where, U is the urban impervious surfaces fraction, V is the vegetation fraction, and C is the composition of the impervious surface inside each pixel. This study used land cover classification result in the first MESMA procedure to analyze the performance of LSTn in different types of land cover. Additionally, we used land cover classification and UIS composition to evaluate our results by measuring the correlation between the two results and LSTn, based on previous studies that noted significant correlations between UHIs, land cover type, and UIS composition [4,12,13,14]. 

### 2.3. Land Surface Temperature Calculation

To estimate the LST based on Landsat thermal infrared data, the digital numbers (DNs) in the thermal bands of Landsat 5 (Band 6) and Landsat 8 (bands 10 and 11) were converted to radiance data using the formula in (Equation (3)) [21,22]

(3) $$L\lambda =\frac{Lmax-Lmin}{Q\mathrm{cal}max-Q\mathrm{cal}min}\times \left(Qcal-Qcalmin\right)+\;LMIN\lambda$$

where the Lλ is the radiance value, Qcalmin is the minimum quantized calibrated pixel value, and Qcalmax is the maximum quantized calibrated pixel value; Lmin is spectral radiance scales to Qcalmin, Lmax is spectral radiance scales to Qcalmax. 

The spectral radiance of each image was converted to brightness temperature values as [21,22]

(4) $$Tk=\frac{K2}{ln\left(\frac{K1}{L\lambda }+1\right)}$$

where Tk is the brightness temperature in Kelvin, Lλ is the spectral radiance value; K1 and K2 are the constants of Landsat calibrations. To convert the brightness temperature from Kelvin to Celsius, expressed as [23]

Tc = Tk − 273.15(5)

where Tc is the brightness temperature in Celsius and Tk is the brightness temperature in Kelvin. Thirdly, LST (in Celsius) was calculated by using the formula in (Equation (6)) [21,22]

(6) $$Ts=\frac{Tc}{1+\left(\lambda \;\times \frac{Tc}{P}\right)\;lnƐ}$$

where Ts is the LST; Tc is the brightness temperature in Celsius; λ is the emitted radiance wavelength; P is the result of (h*c/b) in which h is the Planck’s constant, c is the velocity of light, and b is the Boltzmann constant; and Ɛ is the land surface emissivity (LSE). 

The only missing value in this formula is the LSE. The estimation of LSE starts by calculating the NDVI for reclaimed areas using (Equation (7)) [14,24]

(7) $$NDVI=\;\frac{NIR-RED}{NIR+RED}$$

where NIR is the near infrared band, and RED is the red band in each image. Then, the NDVI results were used to calculate the proportion of vegetation by applying the expression in (Equation (8)) [22,24]

(8) $$Pv=\;\frac{NDVI-NDVImin}{NDVImax+NDVImin}$$

where NDVI*min* is the NDVI minimum value, and NDVI*max* is the NDVI maximum value. The LSE was estimated as [22,25]

(9) $$Ɛ=\left(0.004\times Pv\right)+\;0.986$$

After estimating the LST of all images, seasonal averages were calculated, followed by the overall average of the period LST to be ready for normalization procedure. 

### 2.4. LST Normalization (LSTn)

After estimating LST average for seasonal and over all period data, we applied the LST normalization based on the average of water surface temperature. This normalization process is expressed in the formula

(10) $$n=\frac{X-Wavr}{\mathrm{LST}max-\mathrm{LST}min}$$

where n is the normalized LST (LSTn), X is the LST value, W*avr* is the average value of bay’s water surface temperature, LST*max* is the maximum value of LST, and LST*min* is the LST minimum value within the study area, and the result ratio is varying from −1 to 1. We applied this equation to the estimated average seasonal data for each period, winter, spring/autumn, and summer. The LSTn of all seasons were average to extract LSTn results for each period.

## 3. Results

### 3.1. Land Reclamation Evolution during the Study Period

Based on the water surface of Lingding Bay in 1973, the land reclamation process added about 270 square kilometers to land by 2017 as shown in Table 2. The table shows the evolution of the land reclamation process during the study period and the area of UIS compared to other land cover types. Reclamation added about 60 km^2^ to the coastal lands between 1973 and 1987. In the following 10 years, reclaimed areas increased by about 130 km^2^. The reclamation rates decreased between 1997 and 2007 to 65 km^2^, and continued decreasing and only added 15 km^2^ between 2007 and 2017.

**Figure 4:**
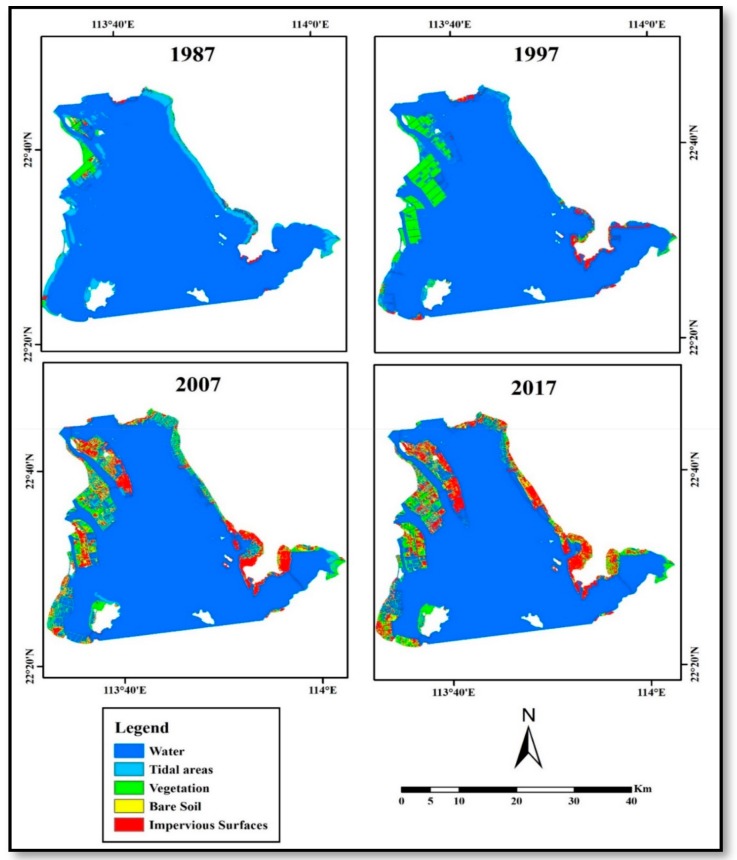
Reclamation process evolution and its land cover classification during the study period.

Urban impervious surfaces (UIS) covered only three square kilometers of reclaimed areas in 1987. UIS area increased rapidly to 21 km^2^, 67 km^2^, and 106 km^2^ of the reclaimed area in 1997, 2007, and 2017 respectively. The distribution of reclaimed areas and their land cover during the study period are illustrated in Figure 4. In 1987, the distribution of reclaimed areas was mainly in the western part of the bay in The Zhongshan City coastal area followed by Guangzhou City, with smaller reclaimed areas in other studied Lingding Bay cities. Land cover in reclaimed areas was mainly vegetation as the reclamation process designed to increase cultivated areas. 

Figure 4 shows an increasing amount of land reclaimed for agriculture purposes in the western part of the bay in 1997. While, the increasing amount of reclaimed areas on the eastern coast in 1997 was mostly UIS. In 2007, UIS continued to expand in the eastern part of the bay, and at the same time, started to increase in the western coast in comparison to previous years. The distribution of land cover in 2017 showed the highest UIS expansion over the study area, thus illustrating the dynamic change in land cover in the reclaimed areas of Lingding Bay over time.

We used the Google Earth™ high spatial resolution images to evaluate land cover classification of 2007 and 2017 periods. We had difficulties to evaluate land cover classification of 1987 and 1997 periods due to the lack of high-resolution images in Google Earth™ at these times. ARCGIS software was used to generate 100 accuracy-sample points based on equalized stratified random sample strategy. This strategy distributes an equal number of points for each type of classification in a random spatial distribution. This verification result revealed an accuracy of 89% and 85% for 2007 and 2017 land cover mapping respectively. The inaccurate classification percentage was due to the mixing between bare soil, and impervious surfaces classification, temporal differences in cultivated areas between vegetation and bare soil land covers, and mixed classification between open water and shallow water classifications, which was differentiated in the coastal area during the process of pixel spectra collection only by the color of the water.

### 3.2. Temporal Changes of LST and LSTn Averages on Reclaimed Areas

**Figure 5:**
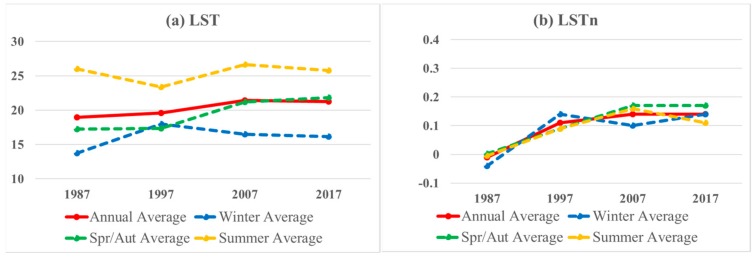
Annual and seasonal averages of (**a**) LST, and (**b**) LSTn.

The annual average trend over the studied years -presented in solid red line in Figure 5 showing an increase in LST from about 19 °C in 1987 to 21.4 °C in 2007, and a slight decrease to 21.3 °C in 2017 as illustrates in Figure 5a. In the same time, LSTn trend shows an increase from −0.01 in 1987 to 0.14 in 2007 and becomes stable in 2017 with same value as shown in Figure 5b. The dashed lines in Figure 5 show the seasonal averages in yellow, green, and blue lines illustrate the seasonal averages for summer, spring/autumn, and winter of LST in Figure 5a, and LSTn in Figure 5b, respectively. 

Figure 5a shows high variance between seasonal LST averages. Seasonal LST differences reached about 10 to 12 °C between summer and winter during the study period, with an exception in 1997. The minimum difference between seasonal averages was recorded in 1997 at only 5.5 °C. However, this small difference is not reliable due to the lack of multiple cloud free images for the winter season in 1997. The only available Landsat image in winter 1997 shows higher LST values compared to common winter season LST averages in the study area. This resulted in an overestimation of the winter season average in 1997 for both LST and LSTn, in contrast to the winter season average in the other studied years. This in turn, resulted in a higher LST winter average in comparison to the spring/autumn LST average as shown in Figure 5a, and a higher LSTn winter average when compared to all other seasonal LSTn averages in the year of 1997 as presented in Figure 5b.

The spring/autumn LST is the only trend that had a continuous increase over the study period from 17.2 °C in 1987 to 21.8 °C in 2017 as shown in Figure 5a. Winter and summer seasonal LST trends fluctuated with a cumulative increasing average of winter seasonal trend of about 3 °C, and an almost stable summer seasonal LST with overall average of about 25.5 °C. On the other hand, LSTn values in Figure 5b, show a narrower range of values for all seasonal trends over the study period, with differences of 0.05 to 0.07, and no discernable differences between the LSTn for the winter season as compared to other LSTn seasonal trends in the year 1997. 

After excluding the LSTn value for 1997, the winter LSTn trend rapidly increased from -0.04 in 1987 to 0.14 in 2017 as illustrated in Figure 5b. The spring/autumn seasonal trend showed increase from 0.002 in 1987 to 0.17 in 2007, and remained in the same value in 2017. Similarly, summer LSTn seasonal trend presented similar increase between 1987 and 2007 from −0.004 to 0.16. However, LSTn summer average decreased to 0.11 only in 2017 as presented in Figure 5b. This decrease was due to the large difference between LST maximum and minimum in 2017; caused by cloud cover in the summer season images that in turn created large gaps in each LST image. Thus, the seasonal LSTn map of 2017 summer combines varied values from different images in one average image. 

### 3.3. LSTn Applicability for Seasonal-Independent Surface Temperature Monitoring

Results of Section 3.2 revealed the applicability of LSTn to reduce seasonal variations of surface temperature in comparison to original LST. In this section, we tested the possibility of selecting cloud free images of different seasons in a single temporal trend to validate the method applicability in cross-seasons cloud free images selection for surface temperature monitoring. The test compared LSTn and regular LST values regarding seasonal limitations. We selected two datasets randomly for this test (test1 and test2), presented in Table 3. Each dataset has a different seasonal representative cloud free cover image for each of the four studied years. Table 3 shows each seasonal representative image, its corresponding season, minimum and maximum LST in the study area and the open water temperature average in the image. 

**Figure 6:**
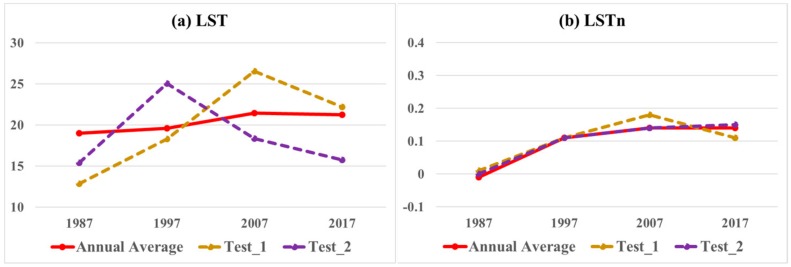
Comparison of original LST and LSTn in temporal analysis with input of different seasonal images.

As shown in Table 3, minimum LST values varied between 12 °C to 22 °C in test1, and 1 °C to about 22 °C in test2. Maximum LST values varied between 19 °C and 34 °C in test1, and from 20 °C to 31 °C in test2. The open water surface temperature averages varied between 15 °C and 24 °C in test1, and 13 °C to 24 °C in test2. We used these seasonal variant of cloud free images to create a surface temperature trend in both LST and LSTn to compare with the extracted average trend of all images that illustrated in Figure 5. Figure 6 shows the comparison of the original LST and proposed LSTn method in accordance to the extracted average of all images in the studied years. 

High variance with distinct seasonal fluctuations as compared to the images average was revealed in the two tested datasets in LST, Figure 6a. Differences were between 5.11 °C and −6.15 °C, the lowest difference value was 0.94 °C. The LSTn in Figure 6b however, shows no difference between the tested images and the overall average in both of the 1997 test datasets or in the 2007 test2 dataset. Variance was limited in LSTn compared to LST values in general with highest difference of 0.04 in 2007 from test1 dataset. Overall, the average of differences revealed from the LSTn was 0.01 only compared to a variance average of 3.89 °C in the original LST. This result shows the advantage of using LSTn method over the original LST data for temporal comparisons and time series analysis in cases of variant seasonal images arising from the absence of free cloud cover images on same dates, seasons, or years.

### 3.4. Land Cover Correlation to LST and LSTn

**Figure 7:**
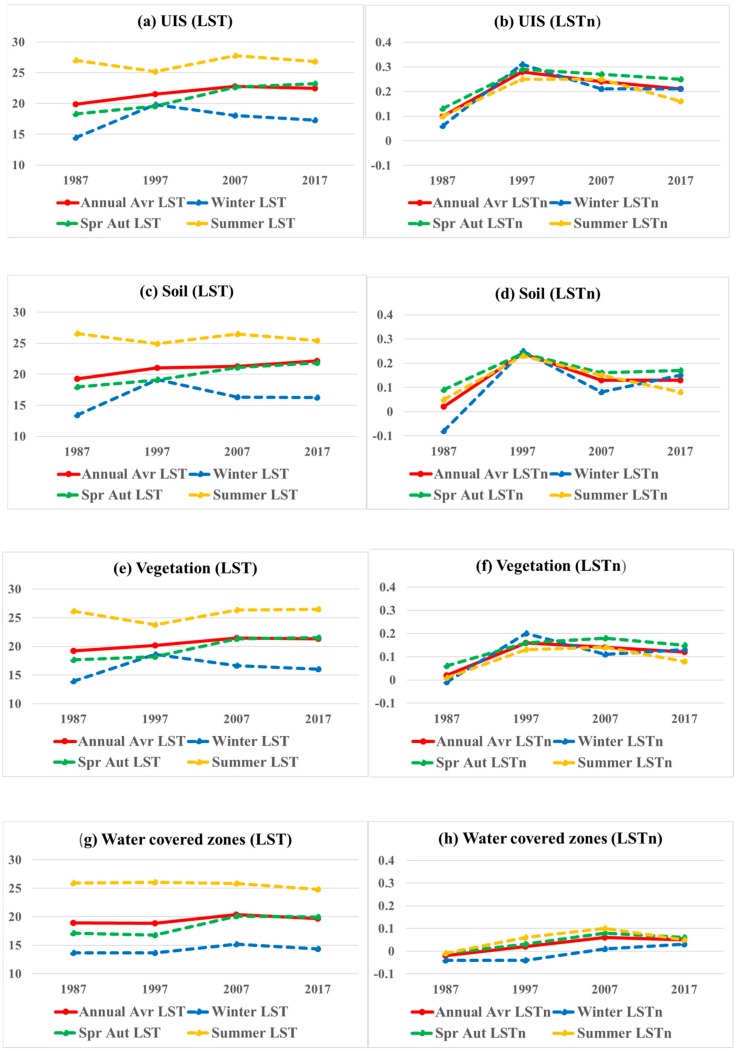
LST and LSTn of UIS, soil, vegetation, and inner water land cover types.

Figure 7 presents LST and LSTn averages based on land cover type. The annual averages of LST and LSTn represented in solid red line of all figures of land cover types. LST averages of all land cover types showed an increase in 2017 in comparison to LST averages in 1987, with small different fluctuations during the study period, as presented in Figure 7a,c,e,g. Similar increases were revealed in annual LSTn trends between 1987 and 2017 as illustrated in Figure 7b,d,f,h, with a remarkably higher annual average in 1997 in UIS, soil, and vegetation land cover types.

As previously mentioned, LSTn reduced the seasonal variance in the original LST, which still can be retrieved from different land cover surfaces, as illustrated in Figure 7. However, caution was needed to take in winter season trends for soil and water covered zones as seen in Figure 7d,h respectively. The winter LSTn on soil shows higher variance from other seasonal trends in 1987 and 2007, and it is same on water covered zones in 1997 and 2007.

### 3.5. Advantage of LSTn for Extraction of Surface Urban Heat Islands (SUHI)

**Figure 8:**
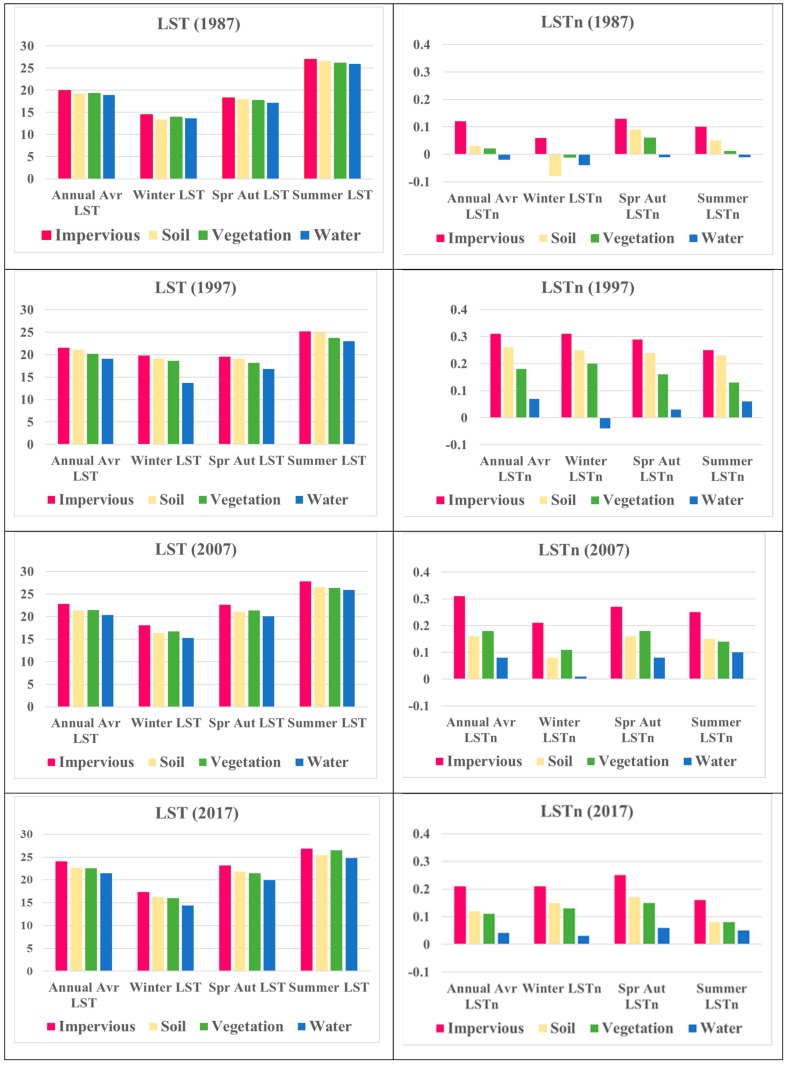
Variation of LST and LSTn based on land cover.

The proposed LSTn method showed higher capability to distinguish urban impervious surfaces (UIS) from other land cover types as showed in Figure 8. This makes the SUHI extraction easier as it is spatially correlated to UIS. The UIS shows significantly higher LSTn values that led to a higher distinguish capability than from other land cover types. Figure 8 presents the differences between LST and LSTn averages for land cover types. LST average of UIS is slightly higher than averages of other land cover types. The close LST averages over land cover types make it difficult to distinguish SUHI spatially. On the other hand, the proposed LSTn method increases the average of UIS compared to other land cover types.

**Figure 9:**
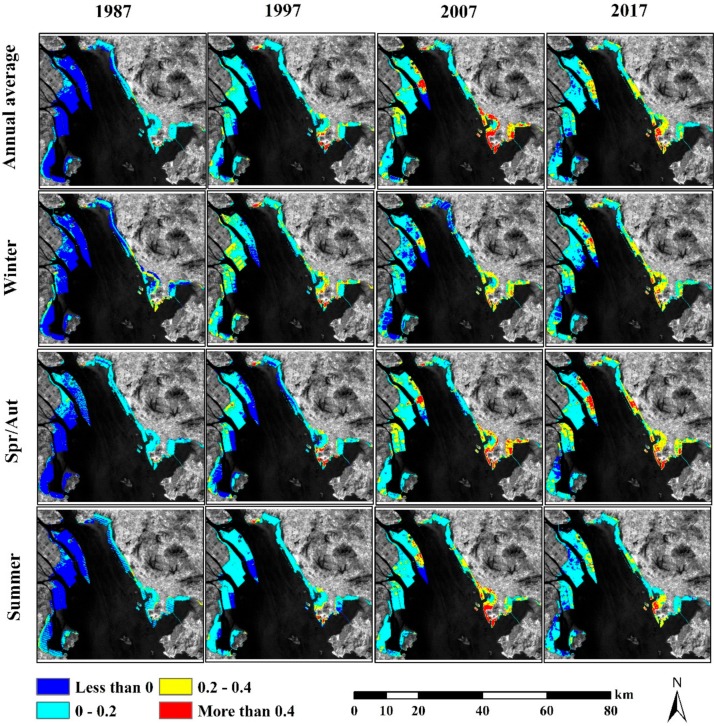
Distribution of annual and seasonal LSTn over the study period.

We used this advantage to map the temporal variation of the SUHI on reclaimed areas of Lingding Bay based on the spatial distribution of LSTn. In this study, we define a threshold of 0.4 for areas of SUHI in Lingding Bay reclaimed lands. This threshold was defined for SUHI extraction based on the highest extracted average of LSTn on UIS land cover of 0.32 in 1997 and 2007. Based on the selected thresholds, SUHI in 1987 exists only small parts in the Shekou Peninsula, the coastal zone of Shenzhen City, and around the Shajiao electric power plant in the northern part of Lingding Bay as illustrated in Figure 9. These two areas were found to have the same SUHI phenomena in winter and the spring/autumn seasons, but at higher influence in the winter. However, the SUHI area decreased in the two areas during summer season.

No additional SUHI areas showed up in 1997. However, the spatial distribution of SUHI on the Shekou Peninsula and around the Shajiao electric power plant was augmented due to the increase in reclaimed land in the economic zone of the Shekou Peninsula, and to the east of the Shajiao electric power plant. The spatial extent of SUHI was seasonally stable in the two areas, with an increase in the LSTn magnitude. The reclaimed areas of the western coast of Lingding Bay showed no SUHI in 1997, as most of these areas were reclaimed for agricultural purposes.

In 2007, the SUHI on Shajiao electric power plant remained similar to 1997 period with a higher impact in the spring/autumn seasons compared to winter and summer seasons. SUHI increased in the reclaimed areas of Shenzhen City in the Lingding Bay coastal area. This increase of SUHI in Shenzhen City coastal area was controlled by the high LSTn magnitude during the summer season, with less impact in spring/autumn seasons, and very low influence in the winter. Additionally, a new SUHI was detected on the Longxie Island at the middle of the Lingding Bay, after the construction of Guangzhou Port and Guangzhou Dock. The SUHI on this part of the Longxie Island was remarkable in the spring/autumn seasons, with less impact in the summer season, and much lower influence in the winter. In 2017, the detected SUHI area was relatively smaller than that of 2007. SUHI was only observed on the reclaimed areas of Shenzhen City. This decrease was controlled mainly by the decrease in LSTn values of the summer season, compared to previous studied years, with relatively smaller change in seasonal SUHI trends of winter and spring/autumn. In addition, a new SUHI was detected at the Bao’an International Airport area in Shenzhen City in the Spring/autumn seasons only. 

## 4. Discussion

LST monitoring supports governmental human and environmental health initiatives and decision-making. SUHI affects the human body, soil moisture, and energy consumption in urban areas and surrounding peri-urban zones [26]. A method for LST comparisons over time, specifically for rapidly changing coastal areas under regular cloudy conditions is urgently needed, despite the limited availability of thermal data due to cloud cover. LST variation over time is always problematic in monitoring applications over time [27]. In this study, we propose a new normalized method to minimize seasonal changes in the LST for a flexible image selection to monitor LST and SUHI over time. LSTn overcomes seasonal and other temporal challenges by normalizing and scaling LST values estimated from Landsat images acquired at different dates, enabling temporal comparisons of surface temperature and extraction of SUHIs over coastal reclaimed areas. 

LST varies widely over space and time; hence, it is difficult to evaluate the accuracy of the LST estimation obtained from Landsat images due to a lack of high-resolution thermal infrared data with identical times of image acquisition. Errors stemming from atmospheric corrections, sensor noise, false land surface emissivity estimation, aerosols and other gaseous absorbers, algorithm processing, sensor view angle effects, and wavelength uncertainty [28,29,30,31], could cascade errors and uncertainty in the LST retrievals. This study used two sets of Landsat sensor data; Landsat5 and Landsat8. On-board calibration of Landsat5 sensor had a consistent offset error of about 0.7 K over the lifetime of the instrument [32]. The uncertainty of Landsat8 TIR laboratory calibration before launch was estimated by about <0.3% at all calibration temperatures for the two TIR bands, with maximum allowed radiance error W/(m^2^ sr µm) of 0.059 (0.4 K at 300 K) for Band 10 and 0.049 (0.4 K at 300 K) for Band 11 [33]. However, the stray light from far out-of-field has influenced the absolute calibration of Landsat8 thermal data since launch. Stray light refers to unwanted radiance from outside the field-of-view recorded by the sensor in the optical system of the scene and could reach rise to about 9 K [34]. Since standard calibration techniques failed to correct for this error, efforts have been made to develop a method for stray light correction in Landsat8 TIR data. The first attempt to fix this error succeeded in reducing the stray light error by 0.29 W/m^2^/sr/um (~2.1 K) in Band 10 and 0.51 W/m^2^/sr/um (~4.4 K) in Band 11. The root-mean-square (RMS) variability was 0.12 W/m^2^/sr/um (~0.8 K) for Band 10 and 0.2 W/m^2^/sr/um (~1.75 K) for Band 11. [34,35]. In this correction effort, stray light reduced extreme errors from 9 K to only about 2 K [34]. In early 2017, Gerace and Montanaro [36], reduced the stray light error from 2.1 K at 300 K to 0.3 K for Band 10 and from 4.4 K to 0.19 K for Band 11, with less variability of 0.52 K at 300 K for Band 10 and 0.91 K at 300 K for Band 11. This final calibration was used in our Landsat8 images, as it was implemented in all Landsat8 products provided by USGS since early 2017 [36].

Jimenez-Muoz and Sobrino [28], concluded the uncertainty of atmospheric effects on thermal remote sensing LST retrieval between 0.2–0.7 K. While estimated the uncertainty of land surface emissivity between 0.2–0.4 K, and concluded a total error of thermal LST ranges between 0.3–0.8 K. Studies with in situ measurements have lower uncertainty values; since we do not have in situ available data, we considered the higher values of uncertainty in our results. Yu et al. [29] compared three different approaches for LST inversion from 41 Landsat8 TIRS images: the radiative transfer equation-based method, the split-window algorithm and the single channel method. The results from this study revealed a small RMSE difference of 0.122 between all processed methods, with an advantage to the radiative transfer equation-based method by RMSE 0.903 K when applied on Landsat8 Band 10. The highest RMSE was 1.67 K for the single channel method when applied on Band 11 of Landsat8 data. Considering all error sources including noise error, bandpass effects, and wavelength indetermination, with no available in situ measurements, we took 0.9 °C as the LST retrieval accuracy of our analysis based on literature [28]. In future work, we plan to measure LST uncertainty and influence on the proposed LSTn method results; and estimate the correlation between uncertainty values and normalized LST values in LSTn. In the current study however, we assume a correlation between LSTn results and land cover, as well as a correlation to the UIS composition as reported in the literature. 

Previous studies revealed a positive correlation between land cover types, mainly UIS and LST [6,7,8,20,21,37]. Therefore, we considered the validation of our proposed method by correlating LSTn to land cover types, and UIS fraction. Land cover types were sorted based on ascending rank as we concluded from the literature, ranked on ordinal values for water (1), vegetation (2), soil (3), and urban impervious surface (4). The Pearson’s correlation coefficient results are revealed in Table 4. All correlations were significant at the 0.01 level for annual and seasonal LSTn results in all studied years. Highest correlation coefficient value for the annual average was presented in 1997 for both land cover classification and UIS fraction. The highest correlation coefficient for seasonal LSTn was varied between winter seasons in 1997 and 2007 for both correlation tests and 1987 UIS fraction test, transitional seasons for both tests in 1997, and land cover classification correlation of 1987. 

To the best of our knowledge, no previous method was conducted to eliminate or reduce the seasonal influence of estimated LST for temporal comparison determination. Our LSTn method yielded a decrease in seasonal variations of surface temperature allowing for improved temporal comparisons independently of seasonal effects. In comparison to the regular LST variations method to detect SUHI, and its correlation to buildings and impervious surfaces [38], the proposed LSTn method revealed sharper edges of surface temperatures between UIS and other land cover types that make SUHIs be more distinguishable in the area of study. 

As mentioned, normalization methods were used previously for surface temperature comparison. Sirous et al. [11] calculated a min-max feature scaling normalized land surface temperature (NLST) to compare their SUHI intensity results between different images that used a different normalization method, but did not consider a specific land cover type for normalization estimation. Amiri et al. [5] used the same normalization method to compare the surface temperature overtime in transitional land cover areas, mainly from vegetation and bare soil to urban impervious surfaces. 

In contrast, our proposed method might consider as a new LST intensity method since it depends on a specific land cover type out of the urban area, as previously estimated in literature based on forest, agriculture areas, and water surfaces [27]. However, this method overcome the uncertainties of urban and surround reference land cover definition as it depends on the average of open water surface temperature as a reference. Nevertheless, this method has some limitations, as it decreases the seasonal influence on the temporal comparison and time series analysis, not removing it permanently, and results could be influenced significantly in case of extreme temperature events such as heat waves in the selected cloud free images. 

## 5. Conclusions

For a long time, the study of temporal LST changes using thermal infrared remote sensing was a matter of identifying similarities during the same season over multiple years. Cloud cover in optical remote sensing added uncertainty and limited the availability of data usage in many areas worldwide. In this paper, we propose a new method for surface temperature monitoring on newly reclaimed areas over time, which is independent from seasonal effects for more flexible optical remote sensing dataset selection over time by normalizing LST data based on the average open water temperature. We used Landsat data at 10-year intervals from 1987 to 2017 to compare LST normalized values (LSTn) over reclaimed areas of Lingding Bay, Southern China.

The LSTn method revealed a relative decrease in seasonal surface temperature variability compared to the original LST results. Hence, the proposed method provides a more robust means to monitor surface temperature over time. LSTn trends had a relatively lower variability average (0.01) to the overall images annual mean in comparison to the LST variability average (3.9 °C). Therefore, detecting of SUHI is easier when using LSTn because it increases urban impervious surface temperature values relative to other land cover types. 

Experiments are required to evaluate the original LST uncertainty influence on LSTn results. Caution is needed when selecting the cloud free images for surface temperature monitoring in cases of extreme temperature events such as strong heat waves in the summer. Additionally, more tests are needed to examine this method for winter surface temperature on soil and water covered zones, as the winter season reveals relatively higher difference to the overall mean images in some studied years in comparison to other studied seasons. 

This method can be applied for coastal urban areas in general because of its dependency on the average of open water temperature for calculating the normalization average. Future work will focus on the influence of estimated LST uncertainty on the normalized results in LSTn, and the applicability of this method in surface temperature comparisons of coastal cities in different climatic zones, based on the nearby open water temperature average. 

## Figures and Tables

**Table 1:** Inventory of the Landsat TM and OLI/TIRs used in the study [17].

Period	Data Type	Season	Date Acquired	Period	Data Type	Season	Date Acquired
1987	TM	Winter	6 Jan. 1987 8 Dec. 1987 * 10 Dec. 1988	2007	TM	Winter	28 Dec. 2006 13 Jan. 2007 29 Jan. 2007 * 17 Dec. 2008
		Spring/Autumn	3 Nov. 1986 24 Nov. 1988			Spring/Autumn	19 Apr. 2007 4 Mar. 2008 23 Sep. 2006 10 Nov. 2006 28 Sep. 2008
		Summer	30 Jul. 1986			Summer	21 Jul. 2006 26 Jul. 2008
1997	TM	Winter	22 Dec. 1998	2017	OLI/TIRs	Winter	7 Feb. 2016 * 7 Dec. 2016 12 Feb. 2018
		Spring/Autumn	3 Mar. 1996 6 Mar. 1997 25 May 1997 30 Nov. 1996 1 Nov. 1997			Spring/Autumn	26 Mar. 2016 30 Apr. 2017 1 Apr. 2018 5 Nov. 2016 23 Oct. 2017
		Summer	9 Jul. 1996 28 Jul. 1997 29 Aug. 1997 * 16 Aug. 1998			Summer	26 Aug. 2017 22 Jul. 2018 07 Aug. 2018

* Images used to extract coastal reclaimed areas and analyzed for land cover classification in each study period.

**Table 2:** Reclaimed land in Lingding Bay since 1973 till 2017.

Year	Reclaimed Area (km^2^)	Land Cover	
		Urban Impervious Surfaces Land Cover (km^2^)	Other Land Cover Types * (km^2^)
**1987**	60	3	57
**1997**	190	21	179
**2007**	255	67	188
**2017**	270	106	164

* Other land cover types include vegetation, soil, and inner water (aqua-agriculture land use and areas remained under reclamation process).

**Table 3:** Images inventory of the two datasets for LSTn seasonal-independent test.

Trend	Period	Image Date		Season	Min. LST (°C)	Max. LST (°C)	Open Water Temp. Avr. (°C)
**Test1**	1987		06.01.1987	Winter	12.34	19.27	15.16
	1997		06.03.1997	Spring	14.7	30.0	16.63
	2007		26.07.2008	Summer	22.36	34.06	24.46
	2017		23.10.2017	Autumn	17.03	32.45	20.54
**Test2**	1987		24.11.1988	Autumn	10.42	20.06	15.41
	1997		29.08.1997	Summer	21.83	31.17	24.05
	2007		04.03.2008	Spring	1.34	28.76	14.41
	2017		12.02.2018	Winter	4.83	24.85	12.75

**Table 4:** Pearson’s correlation coefficient for LSTn to land cover classification and urban impervious surfaces fraction (UIS) *.

Year	Season	Land Cover Classification	UIS Fraction
**1987**	**Annual Average**	0.456	0.443
	**Winter**	0.345	0.423
	**Spring/autumn**	0.410	0.250
	**Summer**	0.334	0.346
**1997**	**Annual Average**	0.711	0.541
	**Winter**	0.571	0.421
	**Spring/autumn**	0.733	0.539
	**Summer**	0.649	0.531
**2007**	**Annual Average**	0.588	0.466
	**Winter**	0.611	0.486
	**Spring/autumn**	0.576	0.461
	**Summer**	0.462	0.356
**2017**	**Annual Average**	0.268	0.329
	**Winter**	0.336	0.389
	**Spring/autumn**	0.307	0.331
	**Summer**	0.162	0.191

* All correlations were significant at the 0.01 level.
